# From Isolation to Compassion: Rethinking Palliative and Dementia Care in Carceral Settings

**DOI:** 10.1093/geroni/igaf122.1033

**Published:** 2025-12-31

**Authors:** Raya Kheirbek, Kenzie Latham-Mintus, Raya Kheirbek

## Abstract

The rapid aging of incarcerated populations presents urgent challenges for correctional institutions, particularly in providing adequate geriatric, palliative, and dementia care. This symposium explores innovative, humane approaches to addressing these needs through collaborative, peer-led, and educational initiatives. Presentations will cover modified research methodologies for conducting usability testing in restrictive environments, the impact of peer-led hospice care on institutional dynamics, the unique challenges faced by individuals with frontotemporal dementia (FTD) in the criminal legal system, and the development of dementia education programs for both correctional staff and incarcerated individuals. By highlighting practical solutions and the transformative potential of compassionate care, this session aims to reframe the conversation around aging and dignity in carceral settings. Incarceration and Aging Interest Group Sponsored Symposium

